# Frequency of Different Types of Diagnostic Errors in Patients with Central Nervous System Infections: A Cross-Sectional Observational Study

**DOI:** 10.1155/2018/4210737

**Published:** 2018-11-19

**Authors:** HamidReza Naderi, Fereshte Sheybani, Omid Khosravi, Mehdi Jabbari Nooghabi

**Affiliations:** ^1^Department of Infectious Diseases and Tropical Medicine, Faculty of Medicine, Mashhad University of Medical Sciences, Mashhad, Iran; ^2^Imam Reza Clinical Research Unit, Faculty of Medicine, Mashhad University of Medical Sciences, Mashhad, Iran; ^3^Faculty of Medicine, Mashhad University of Medical Sciences, Mashhad, Iran; ^4^Department of Statistics, Faculty of Mathematical Sciences, Ferdowsi University of Mashhad, Mashhad, Iran

## Abstract

**Objectives:**

To assess the frequency of different types of diagnostic errors in patients with central nervous system (CNS) infection from the onset of symptoms to admission to the hospital, where the correct diagnosis was made.

**Methods:**

A cross-sectional observational design was used, and the information was collected by interviewing patients and/or their knowledgeable relatives as well as reviewing the accompanying medical record documents and hospital records.

**Results:**

Of 169 adult patients with CNS infection, 129 (76.33%) were subject to diagnostic errors. Failure in ordering tests and hypothesis generation were the most common types of diagnostic errors that accounted for more than 70% of errors. Several contributing factors that were associated with incorrect diagnostic hypotheses included failure in taking a patient's comprehensive history such as detecting relevant epidemiological clues, conducting a full clinical examination, and interpreting diagnostic evidence. The relationship between poor clinical outcome and longer delay from the onset of illness to diagnosis, inappropriate empirical antibiotic therapy, and lower level of consciousness on admission were found to be statistically significant.

**Conclusions:**

Although diagnosis and management of CNS infection in some patients are straightforward, clinical decision making in facing patients with complex scenarios often requires clinical reasoning instead of relying only on intuitive diagnosis. Justification in requesting diagnostic measures and interpretation of their results based on clinical findings and patient information could be a critical factor in preventing a substantial number of diagnostic errors in patients with CNS infection.

## 1. Introduction

Healthcare system is an industry prone to accidents. Although the introduction of complicated modern diagnostic and therapeutic measures has improved patient care, it has also increased the probability of accidents and other unfortunate events which may harm the patient [1]. There is no global accurate estimate of such errors; however it shows an increasing trend [2, 3]. A substantial number of patients worldwide continue to be harmed while receiving care [4]. In classifying most common causes of death, “medical error” is ranked among the top ten issues [3, 5]. Therefore, patient safety has attracted considerable international attention in the last decade [6–8].

According to the classification of medical errors, presented by the Institute of Medicine, there are four types of errors including diagnostic-, treatment-, preventive-related, and other [9]. Failure in diagnostic process is an important category of medical errors. Although it is difficult to roughly calculate the current rate of diagnostic errors, it has been estimated at 10-15% [10]. This type of error occurs in all specialized fields, especially those that involve high level of stress, work load, and lack of concentration. Diagnostic errors are also more likely to occur when the level of uncertainty is high, such as when the physician is unfamiliar with the disease, or when the presentation of an illness is atypical or nonspecific, or in the confusing situations [11–13].

Central nervous system (CNS) infection is a medical emergency that may be associated with significant morbidity and mortality, often necessitating emergent diagnosis and treatment [14]. Missed or delayed diagnosis of CNS infections can lead to devastating consequences for patients, health professionals, and the healthcare system [15–17]. There are a number of case reports and series found in the literature regarding errors in diagnosis of CNS infections [18–22].

Although intracranial infections such as meningitis have been reported among the most frequently investigated conditions involved in diagnostic error or delay [23, 24], our literature review did not find any investigation focusing on the root cause analysis of diagnostic errors in patients with CNS infections. The objective of this study was to investigate different types of errors in the diagnosis of CNS infections from symptoms onset to diagnosis. We also discussed the factors contributing to these errors.

## 2. Materials and Methods

This study was a cross-sectional investigation conducted in Imam Reza Teaching Hospital affiliated to Mashhad University of Medical Sciences, Mashhad, Iran, from July 23, 2015, to August 22, 2017.

All hospitalized adult patients with final diagnosis of CNS infection were enrolled consecutively. In the setting of appropriate clinical syndromes, CSF analysis and appropriate microbiological and imaging studies were used for confirmation of the diagnosis of CNS infection. Exclusion criteria included patient unwillingness to participate in the study.

Patient's information, including demographic, clinical, and para-clinical data, history of medical visits, diagnostic and therapeutic managements, referrals to the healthcare systems, and other related data, was recorded. The information was collected by interviewing patients and/or their knowledgeable relatives as well as reviewing the accompanying medical record documents. In the next stage, all the retrieved data were assessed by two infectious diseases specialists to detect the diagnostic errors and determine their possible explanations. Then, the data were classified based on the “Diagnostic Error Evaluation and Research (DEER) Taxonomy Chart Tool” first introduced by Schiff et al. [12].

### 2.1. Definitions

#### 2.1.1. Diagnostic Error

A diagnostic error was defined as a misdiagnosis or a delay in the diagnosis of CNS infections.

#### 2.1.2. Incomplete History Taking

It was defined by not paying attention to patient's key complaints, risk factors, and previous contact histories, as well as epidemiologic clues.

#### 2.1.3. Incomplete Physical Examination

It was defined by failure to discover the clinical signs related to patient's illness, especially not paying attention to several key elements of physical examination such as vital signs, testing the meningeal signs, or assessing the neurological deficits.

#### 2.1.4. Improper Referral

It was defined as a failure to refer a patient for emergency department in a timely manner based on the patient's history, or as a referral to an inappropriate center.

#### 2.1.5. Major Sequelae

It referred to an overt functional disability at the time of discharge from hospital that was detectable by clinical examination, not including subtle neurological deficits such as audiometric hearing impairment.

#### 2.1.6. Polypharmacy

It was defined as prescription of multiple inappropriate medications, including antimicrobials, corticosteroids, or analgesics.

### 2.2. Statistical Methods

Data are presented as number (percentage) or mean ± standard deviation. Chi-squared, maximum likelihood ratio, or Fisher exact tests were used in a contingency table to investigate the hypotheses of this study. A P-value <0.05 was considered as the significance level.

### 2.3. Ethical Considerations

This research has been approved by the Committee on Ethics of Mashhad University of Medical Sciences with the IR.MUMS.fm.REC.1394.500.

## 3. Results

**Figure 1:**
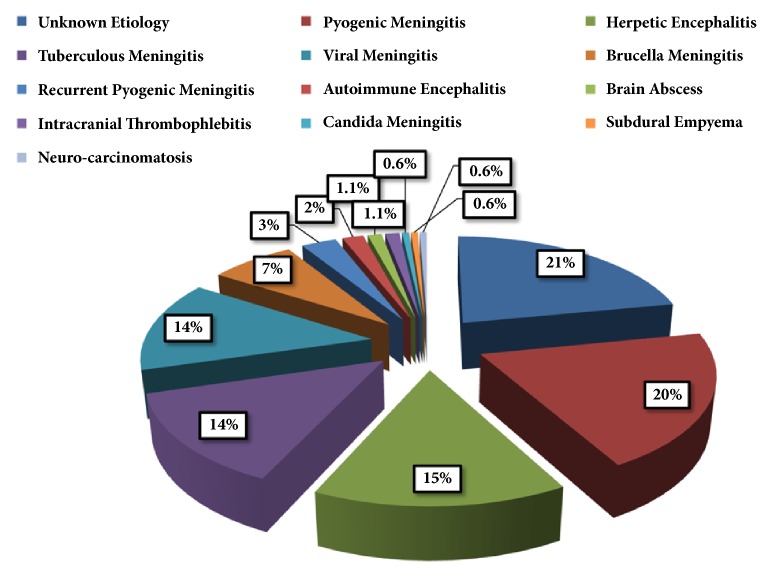
The frequency distribution of CNS involvement and other non-infectious meningoencephalitis/encephalitis syndromes in relation to different etiologies.

There were 169 patients with the mean age of 59.88 ± 16.38 years (15 to 90 years). Ninety-five subjects were male (56%) and 74 were female (44%). The frequency distribution of CNS involvement and other non-infectious meningoencephalitis/encephalitis syndromes in relation to different etiologies is illustrated in Figure 1. Although, our objective was to investigate diagnostic errors in CNS infections, three cases of autoimmune encephalitis and one case of neuro-carcinomatosis were not omitted, because these cases were also manifested as meningoencephalitis, and the diagnostic errors in the course of their illnesses were relevant to the survey objective.

Upon admission to the hospital, 14 (8%) patients developed major sequelae, and seven (4%) died. Of the seven patients who died, three had pyogenic meningitis, and three other subjects were diagnosed as having neuro-carcinomatosis, herpetic encephalitis, and tuberculous meningitis. For one patient, the etiologic diagnosis of CNS involvement remained unknown.

Table 1 shows the frequency distribution of clinical outcome in relation to several variables. It illustrates that the only variables that showed a significant relationship with poor clinical outcome were the longer delay in time to diagnosis from the onset of illness, inappropriate antimicrobial prescription, and lower level of consciousness, based on GCS score on admission.

It demonstrates that inappropriate prescription of antibiotics (with presumed diagnosis of sinusitis or an unspecified illness) and steroids (for symptomatic relief of headache) before making the correct diagnosis occurred in 73% and 54% of the patients, respectively. Table 1 also demonstrated that, of the 117 patients in whom no suspicion of CNS infection was stated in previous medical visits, nine (8%) developed major sequelae, and 5 (4%) died. In other words, for 71% of 126 patients with major sequelae and for 79% of 63 subjects who died, meningoencephalitis was not a clinical diagnosis prior to hospital admission.

**Figure 2:**
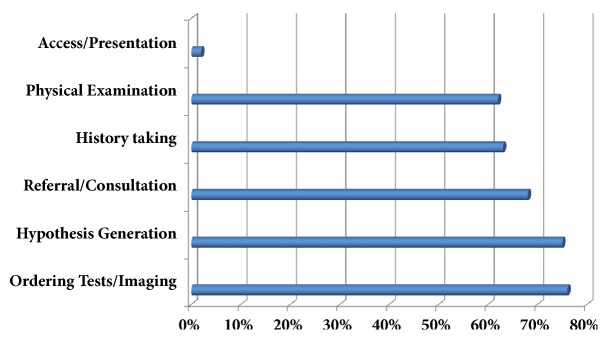
The frequency distribution of diagnostic errors in patients with CNS infection, from the first visit by a physician to hospitalization.

The frequency distribution of different types of diagnostic errors from symptom onset to diagnosis is shown in Figure 2. The most prevalent types of error include failure in ordering tests (i.e., failure to request appropriate laboratory exams (such as lumbar puncture) and/or brain imaging well-timed) in 129 (76.33%) cases; failure in hypothesis generation (i.e., failure or delay in considering the diagnosis as a result of misperceiving, misreading, or misinterpreting the evidence), referral/consultation (i.e., failure or delay in referring the patient to an appropriate center/specialist and in requesting justified specialty consultation), physical examination (i.e., failure or delay in detecting critical physical examination findings such as meningeal sings), and history taking (i.e., failure or delay in detecting a critical element of history data); and failure in access/presentation (i.e., not having access to a primary care center or not presenting to a physician in a timely manner), respectively.

## 4. Discussion

Our study highlighted several important concerns: first, a high rate of diagnostic errors in patients with CNS infections [129 (76.33%) cases]; second, rooting out the failure in ordering appropriate tests/imaging, hypothesis generation, and history taking (76.3%, 75.1%, and 62.1% of patients, respectively) as the main types of error responsible for missed or delayed diagnosis along with failure in physical examination and referral/consultation; third, prescription of inappropriate polypharmacy including corticosteroids to more than half of patients before arrival to the hospital, where the correct diagnosis was made; finally, a significant association of patients' poor clinical outcome with lower level of consciousness on admission, inappropriate antimicrobial prescription, and the longer interval from symptom onset to diagnosis, especially in patients whose diagnosis was delayed by more than three weeks after the onset of illness.

CNS infection presents a unique challenge to physicians because of the potential morbidity and mortality that they cause and also the difficulties involved in their treatment [25]. Early diagnosis and prompt treatment are the mainstay of their successful management. As previously demonstrated in patients with bacterial meningitis, any delay in diagnosis and antimicrobial treatment after patient arrival in the emergency department was associated with adverse clinical outcomes, especially when the patient's condition advanced to a high stage of severity [8, 26]. Here, we try to discuss several potential contributing factors for presentation of a patient with CNS infection in advanced stages of severity after a considerable delay in diagnosis, based on our findings and available information in the literature.

As demonstrated by the present study, one of the main type of diagnostic error was failure to generate an appropriate hypothesis from the patient information. For example, while it has been suggested that any abnormal behavior should be considered as infectious encephalitis [27] until proven otherwise, about 30% of our patients with encephalitis were referred to a psychiatric hospital with the assumption that the illness originates from some psychological condition. Some of the possible contributing factors for the shortcomings in hypothesis generation in patients with CNS infection are as follows: (1) a high rate of failure in obtaining accurate history and performing physical examination as demonstrated in our patients; (2) premature closure of diagnosis which means stopping the diagnostic process, assuming the first diagnosis is correct, and failing to consider other possible differential diagnoses [28] as occurred in a significant proportion of our patients (for better clarification, Table 2 provides a few examples of diagnostic errors committed on the patients); (3) the presence of classic triad of fever, neck stiffness, and altered mental status in only less than half of meningitis patients [14] that could be one of the possible reasons for failure in hypothesis generation of meningitis despite complete physical examination [19]; (4) although in medical education programs, significant emphasis is placed on clinical clues suggestive of headaches secondary to subarachnoid hemorrhage and also brain tumors, headaches secondary to infectious etiologies are often overlooked [17]; (5) another potential underlying cause of delayed diagnosis in our patients might be high rate of inappropriate polypharmacy before making the definitive diagnosis because the effect of some medications such as corticosteroids and antibiotics could mask or change the typical symptoms and signs of CNS infection.

Additionally, while the diagnosis of meningitis rests on CSF examination by LP, sometimes physicians postpone the procedure by requesting a few futile diagnostic measures [15] that could be associated with poor outcome. For example, it has been shown that the time involved in waiting to undergo brain CT scan significantly delays the initiation of antimicrobial therapy, with the potential for increased morbidity and mortality in patients with bacterial meningitis [15]. Therefore, it should be avoided in the situations that there is no indication for neuroimaging before LP [29].

We also found that errors are frequently associated with misinterpretation of a para-clinical test result, sometimes with definite indication and sometimes without indication. For instance, more than 70% of the patients in our study were discharged from the medical centers despite complaining of excruciating or unexplained headache and other suggestive symptoms, because their brain CT scans were normal. Accordingly, justification in requesting diagnostic measures and interpretation of their results based on clinical findings and patient information could be a critical factor in preventing some important diagnostic (and also other types of medical) errors.

Our study had some strength as well as limitations. As a strength, the study was an analysis of different types of diagnostic errors in CNS infection. The analysis of incidents is a powerful approach of detection of medical errors in order to improve patients safety by adopting protocols or changes in the field where they are more accident-prone [30]. However, there were several limitations: firstly, the possibility of hindsight bias and overestimation of diagnostic errors; secondly, the possibility of underestimation of errors because some patients might have died before the verification of the diagnosis; thirdly, the chance of patients forgetting the course of illness and medical visits; finally, since this study was conducted in only one referral teaching hospital, it might not be generalizable to all patients with CNS infections.

## 5. Conclusions

The present investigation highlighted the high rate of diagnostic errors in patients with CNS infections and the influence of these errors on poor clinical outcome. Several factors were associated with incorrect diagnostic hypotheses, which included failure in taking a patient's comprehensive history as well as detecting relevant epidemiological clues, conducting a full clinical examination, and interpreting diagnostic evidence. Covering up symptoms while ignoring their underlying causes often delays a diagnostic process or obscures the typical presentation of the diseases. We found a significant relationship between poor clinical outcome and lower level of consciousness on admission, inappropriate antimicrobial therapy, and the longer interval from the onset of illness to diagnosis. Our study reemphasizes the importance of using the patient history and physical examination as a basis for selecting relevant diagnostic testing and interpretation of their results in the context of clinical findings and patient information, which can lead to a timely and accurate diagnosis.

## Figures and Tables

**Table 1:** The frequency distribution of clinical outcome in relation to several variables.

**Parameter**		**Complete recovery**	**Sequelae**	**Dead**	**P value**
**Symptom onset to diagnosis**	**< 1 week**	78 (91.5%)	5 (6%)	2 (2.5%)	< 0.001
	**1-3 weeks**	55 (100%)	0	0	
	**> 3 weeks**	15 (51.5%)	9 (31%)	5 (17.5%)	
**No. of prior visits**	**Once**	60 (87%)	5 (7.5%)	4 (5.5%)	0.634
	**More than once**	88 (88%)	9 (9%)	3 (3%)	
**Incomplete history taking**	**Yes**	93 (88.5%)	9 (8.5%)	3 (3%)	0.571
	**No**	55 (86%)	5 (8%)	4 (6%)	
**Incomplete physical examination**	**Yes**	92 (88.5%)	8 (7.5%)	4 (4%)	0.908
	**No**	56 (86%)	6 (9%)	3 (5%)	
**Failure in referral**	**Yes**	48 (86%)	4 (9%)	1 (5%)	0.542
	**No**	100 (90.5%)	10 (7.5%)	6 (2%)	
**Receiving antibiotics before diagnosis of CNS infection**	**Yes**	112 (91%)	9 (7.5%)	2 (1.5%)	0.030
	**No**	36 (78%)	5 (11%)	5 (11%)	
**Receiving corticosteroids before diagnosis of CNS infection**	**Yes**	76 (84.5%)	9 (10%)	5 (5.5%)	0.391
	**No**	72 (91%)	5 (6.5%)	2 (2.5%)	
**Considering CNS infection **	**Yes**	45 (86.5%)	5 (9.5%)	2 (4%)	0.913
	**No**	103 (88%)	9 (8%)	5 (4%)	
**GCS level on admission**	**< 12**	15 (52%)	9 (31%)	5 (17%)	< 0.001
	**12-14**	81 (92%)	5 (6%)	2 (2%)	
	**15**	52 (100%)	0	0	

CNS: Central nervous system; GCS: Glasgow Coma Scale.

**Table 2:** Examples of diagnostic errors based on cognitive contributions to error.

**Cases**	**Type of error**
An 18-year-old girl was scheduled to sit her university entrance examinations in a couple of weeks while presented to the emergency department with acute onset of aggression and abnormal behavioral. The emergency physician diagnosed her as suffering from a hallucination disorder due to exam stress and—ignoring the patient's high fever—referred the patient to a psychiatric hospital. It was ten days until they noticed her unusually high fever and referred her to the infectious diseases ward. The patient was diagnosed with herpetic encephalitis, but she was discharged from hospital with severe sequelae.	Premature closure of diagnosis

A 28-year-old, drug-addicted prisoner was taken to the prison medical clinic by his roommate because of loss of consciousness. The physician's first probable diagnosis was narcotics abuse, and the young man was sent to the city hospital. The neurologist confirmed this diagnosis—without examining the patient—and referred him to a tertiary hospital, where he was hospitalized in the ICU. A post-mortem autopsy proved acute bacterial meningitis.	Premature closure of diagnosis

A 22-year-old boy visited his otolaryngologist with complaint of nasal watery discharge. The symptom began 3 days after nasal polyp removal. The doctor prescribed antihistamines for him. Two days later, he was referred again with severe headache and fever, but the doctor only prescribed cefixime and ibuprofen. The night after this visit, he was brought to the emergency department with agitation and high-grade fever. Lumbar puncture revealed bacterial meningitis and the patient died a few hours later.	Misjudging the salience of findings

A 63-year-old man with gastric lymphoma presented to an emergency unit with complaint of vomiting and severe headache. Frequent vomiting caused him heartburn and the clinical impression assumed to be an ischemic heart attack. But he was discharged because of normal ECGs and serum cardiac enzymes. The day after, he was brought to our emergency department with febrile encephalopathy. On examination, he had decreases level of consciousness and meningeal signs. Lumbar puncture revealed bacterial meningitis. He became intubated and after 21 days of admission in intensive care unit, he was discharged from hospital with severe sequelae.	Faulty perception

A 26-year-old girl was admitted to a hospital with complaint of fever and headache 3 weeks ago. The day after her labs were drawn, she was referred to a nephrology center because of severe hyponatremia. The cranial nerve palsies which were subtle at admission progressed and level of consciousness decreased during the following week. After 10 days a consultation with infectious diseases specialist was requested due to her continuous fever. On examination, she had stiff neck and positive Kernig's sign. Brain CT scan showed hydrocephalus and chest X-ray illustrated a miliary pattern. She was transferred to infectious diseases ward with the diagnosis of tuberculous meningitis/disseminated tuberculosis, but she never recovered completely.	Misjudging the salience of findings

## Data Availability

The data used to support the findings of this study are included within the article.
